# Docosahexaenoic Acid Suppresses Expression of Adipogenic Tetranectin through Sterol Regulatory Element-Binding Protein and Forkhead Box O Protein in Pigs

**DOI:** 10.3390/nu13072315

**Published:** 2021-07-05

**Authors:** Jui-Ting Yang, Yu-Jen Chen, Chao-Wei Huang, Ya-Chin Wang, Harry J. Mersmann, Pei-Hwa Wang, Shih-Torng Ding

**Affiliations:** 1 Department of Animal Science and Technology, National Taiwan University, Taipei 10672, Taiwan; r99626022@ntu.edu.tw (J.-T.Y.); d01642005@ntu.edu.tw (Y.-J.C.); d98626004@ntu.edu.tw (C.-W.H.); b90606041@ntu.edu.tw (Y.-C.W.); mersmann@msn.com (H.J.M.); demonwang@ntu.edu.tw (P.-H.W.); 2 Institute of Biotechnology, National Taiwan University, Taipei 10672, Taiwan; 3 Department of Tropical Agriculture and International Cooperation, National Pingtung University of Science and Technology, Pingtung 91201, Taiwan

**Keywords:** tetranectin, docosahexaenoic acid (DHA), forkhead box O protein (FoxO), sterol regulatory element-binding protein-1c (SREBP-1c), adipose tissues, pigs

## Abstract

Tetranectin (TN), a plasminogen-binding protein originally involved in fibrinolysis and bone formation, was later identified as a secreted adipokine from human and rat adipocytes and positively correlated with adipogenesis and lipid metabolism in adipocytes. To elucidate the nutritional regulation of adipogenic TN from diets containing different sources of fatty acids (saturated, *n*-6, *n*-3) in adipocytes, we cloned the coding region of porcine TN from a cDNA library and analyzed tissue expressions in weaned piglets fed with 2% soybean oil (SB, enriched in *n*-6 fatty acids), docosahexaenoic acid oil (DHA, an *n*-3 fatty acid) or beef tallow (BT, enriched in saturated and *n*-9 fatty acids) for 30 d. Compared with tissues in the BT- or SB-fed group, expression of TN was reduced in the adipose, liver and lung tissues from the DHA-fed group, accompanied with lowered plasma levels of triglycerides and cholesterols. This in vivo reduction was also confirmed in porcine primary differentiated adipocytes supplemented with DHA in vitro. Then, promoter analysis was performed. A 1956-bp putative porcine TN promoter was cloned and transcription binding sites for sterol regulatory-element binding protein (SREBP)-1c or forkhead box O proteins (FoxO) were predicted on the TN promoter. Mutating binding sites on porcine TN promoters showed that transcriptional suppression of TN by DHA on promoter activity was dependent on specific response elements for SREBP-1c or FoxO. The inhibited luciferase promoter activity by DHA on the TN promoter coincides with reduced gene expression of TN, SREBP-1c, and FoxO1 in human embryonic kidney HEK293T cells supplemented with DHA. To conclude, our current study demonstrated that the adipogenic TN was negatively regulated by nutritional modulation of DHA both in pigs in vivo and in humans/pigs in vitro. The transcriptional suppression by DHA on TN expression was partly through SREBP-1c or FoxO. Therefore, down-regulation of adipogenic tetranectin associated with fibrinolysis and adipogenesis may contribute to the beneficial effects of DHA on ameliorating obesity-induced metabolic syndromes such as atherosclerosis and adipose dysfunctions.

## 1. Introduction

Obesity is a worldwide prevalent epidemic and, in recent times, has arisen to a global all-time high. The prevailing overweight/obesity in the human population is evidenced in both developed and developing countries [1]. Moreover, obesity affects approximately over 60% of the US adult population, who are classified as either overweight or obese [2,3]. Obesity plays a causal role in the pathogenesis of metabolic diseases and cardiovascular diseases. In addition, dysfunction of adipose tissues raises the risk of obesity-related metabolic syndromes such as impaired glucose metabolism, insulin resistance, hypertension, elevated blood pressure, hypertriglyceridemia, and dyslipidemia [4].

Tetranectin (TN) is a homotrimeric protein originally found in human plasma during purification of α2-plasmin inhibitor [5]. It is identified as a C-type lectin protein, encoded by the *CLEC3B* gene (C-type lectin domain family 3, member B) [6]. Although the biological functions of TN still are not fully uncovered, TN has a wide variety of roles in fibrinolysis [7], bone formation including mineralization [8,9], and cancer progression and metastasis [10,11,12,13,14]. Tissue expression of TN is prevalent and detectable in various tissues, including heart, brain, lung, liver, skeletal muscle, kidney and pancreas tissues, with the highest levels in lungs [15].

Docosahexaenoic acid (DHA), an *n*-3 polyunsaturated fatty acid (PUFA), has been shown to reduce obesity-related metabolic syndromes [16]. Plasma triglycerides and the adipose tissue mass are reduced by dietary PUFA; *n*-3 PUFA promotes lipolysis by binding to several distinct nuclear receptors including peroxisome proliferator activated receptors (PPAR) α, β/δ, and γ, sterol regulatory element binding proteins (SREBP) and liver X receptors (LXR) [17]. DHA acts as a signaling molecule, and interacts with several transcription factors and nuclear receptors that are involved in adipocyte differentiation and inflammation including CCAAT/enhancer-binding proteins (C/EBP), SREBP, PPAR, LXR and forkhead box O proteins (FoxO) [18,19]. However, the role and underlying mechanism of DHA on TN expression in lipid metabolism remains unclear.

From our preliminary results, TN is an adipokine secreted by human adipocytes; it is increased during adipogenesis of human adipocytes, positively correlated with the body mass index of clinical subjects and upregulated by high-fat feeding in mice [20]. Therefore, the aim of this study was to investigate the nutritional modulation of TN expression by DHA in animal models and human cell lines. Furthermore, promoter analysis was employed to identify putative binding sites for transcription factors on the TN promoter that are regulated by DHA supplementation. 

## 2. Materials and Methods

### 2.1. Animal Study

All the animal experiments were approved by the Institutional Animal Care and Use Committee (NTU-102-EL-3) at National Taiwan University (NTU), Taiwan. Thirty crossbred (Landrace x Yorkshire x Duroc) weaned pigs (mean weights = 18.4 ± 0.3 kg) were obtained from a commercial farm and maintained at the experimental pig farm in the Department of Animal Science, NTU. The diet formula, enriched in saturated/*n*-9, *n*-6, or *n*-3 fatty acids, was chosen for weaned pigs according to our previous study [19]. Briefly, pigs were supplemented with either 2% (as-fed basis) beef tallow (BT), soybean oil (SB), or DHA oil (DHASCO, DSM Nutritional Products, Heerlen, The Netherlands) for 30 d with diets and water provided ad libitum. After 30 d, pigs were sacrificed by electrical stunning coupled with exsanguination, and subcutaneous adipose tissues (dorsal regions), lung and liver tissues were snap-frozen in liquid nitrogen and stored at −70 °C until use.

### 2.2. Cell Culture and Adipocyte Differentiation from Porcine Adipose Stem Cells

Detailed procedures for isolating porcine adipose-derived stem-cells (pADSC)/preadipocytes within the stromal-vascular fraction and differentiation into mature adipocytes were described previously [21,22]. Briefly, apart from in vivo study, additional male piglets (Landrace x Yorkshire) at the age of 5–9 d were purchased from a local pig farm and sacrificed by electrical stunning coupled with exsanguination. The dorsal region of back fat (subcutaneous adipose tissues) was collected in Dulbecco’s Modified Eagle Medium (DMEM; Thermo Scientific, Waltham, MA, USA), sliced using a tissue slicer, and minced under sterile conditions. pADSC were harvested after digestion (600 IU per g of fat per ml DMEM) with collagenase (C6885, Sigma-Aldrich, St. Louis, MO, USA) at 37 °C, red-blood cells lysis and several serial washing steps. Isolated pADSC were seeded and maintained in DMEM/F12 (Thermo Scientific) with 10% fetal bovine serum (FBS; Biological Industries, Beit-Haemek, Israel) and 1% antibiotics/antimycotics (penicillin (100 U/mL), streptomycin (0.1 mg/mL), and amphotericin B (0.25 μg/mL)) (Biological Industries) at 37 °C in an incubator with 5% CO_2_ in air. Then, confluent pADSC were differentiated into mature adipocytes with a chemical induced cocktail (insulin, 3-isobutyl-1-methylxanthine, dexamethasone, transferrin, T3, rosiglitazone) in the basal medium DMEM/F12 containing 1% antibiotics/antimycotics. After 9 d differentiation, at least 70% of pADSC were differentiated into mature adipocytes with apparent lipid accumulation. The HEK293T cells, a human embryonic kidney cell line derived from ATCC, were maintained in DMEM with 10% FBS and 1% antibiotics/antimycotics. Before fatty acid treatments, mature adipocytes and HEK293T cells were serum-starved for 12 h and then supplemented with DHA or palmitic acids (PA) (Cayman Chemical, Ann Arbor, MI, USA) for 24 h. Fatty acids (DHA or PA, dissolved in ethanol) were conjugated with fatty acid-free bovine serum albumin (BSA; US Biological, Salem, MA, USA) at a molar ratio of 4:1 before being delivered to the cells. An equal amount of ethanol coupled with BSA was supplemented in the control group as vehicle controls.

### 2.3. Cloning Porcine TN cDNA

The coding sequence (CDS) of tetranectin (TN, also *CLEC3B*) was cloned from the cDNA library of porcine lungs. Primers were designed according to predicted TN sequence annotated by automated computational analysis of the genomic sequence from the NCBI database, and they are listed in Table 1. PCR products containing the partial CDS of TN were amplified using the Phusion High-Fidelity DNA Polymerase (Thermo Scientific) as follows: 98 °C for 3 min followed by 39 cycles of 98 °C for 10 s, 58–60 °C (for each specific primer set in Table 1) for 30 s and 72 °C for 40 s with a final extension step at 72 °C for 10 min. Partial cDNA sequences of porcine TN were amplified again by the PCR reaction to obtain the full TN CDS. PCR products were then separated by gel electrophoresis, purified with a gel elution kit (Genemark, GMbiolab Co. Ltd., Taichung, Taiwan) and cloned into pJET1.2 cloning vectors (CloneJET PCR Cloning Kit, Thermo Scientific). Plasmids were subsequently sequenced to confirm the cloned DNA sequence of TN. The homology of porcine CDS of TN was compared to other species using the ClustalW or NCBI BLAST program.

### 2.4. Cloning the Putative Porcine TN Promoter

Porcine genomic DNA (gDNA) was isolated to clone promoter regions of porcine TN. Primers were designed according to the gDNA sequence within the CDS upstream of TN, located on porcine chromosome 13, from the NCBI database (Table 2), and the annealing temperature for all primers was 60 °C in PCR reactions (Phusion High-Fidelity DNA Polymerase, Thermo Scientific). A 1956-bp portion of the porcine TN promoter (wild-type (WT)-S1) was cloned into pJET1.2 cloning vectors. Plasmids were subsequently sequenced to confirm the cloned DNA sequence of the TN promoter. The homology of the porcine TN promoter was compared to other species using the ClustalW or NCBI BLAST programs. Putative binding sites for transcription factors were predicted by the online website TFsitescan. One sterol regulatory element (SRE) for sterol regulatory element-binding transcription factor 1c (SREBP-1c) and two forkhead response elements (FHRE) for FoxO were predicted to be present on the porcine TN promoter. Then, two clones with a mutated binding site (−759 to −766) for the SRE of the SREBP-1c, two clones with a first binding site (−1251 bp to −1256) for the forkhead response elements (FHRE) of the FoxO, and two clones with a mutated second binding site (−642 bp to −647) of FHRE for FoxO were cloned by designed primers (Table 2). First, PCR reactions were set at 98 °C for 3 min followed by 39 cycles of 98 °C for 10 s, 60 °C for 30 s and 72 °C for 80 s with a terminal extension at 72 °C for 10 min. Second, partial promoter sequences (first and second half part) of porcine TN were amplified again by the PCR reaction to obtain the full length of mutated TN promoter with each individual mutation. Finally, promoter regions of porcine TN from PCR products of WT or mutated clones were individually cloned into the pGL3-Basic vector of firefly pGL3 luciferase reporter vectors (Promega, Madison, WI, USA) with cutting sites of HindIII and KpnI (Fermentas restriction enzymes, Thermo Scientific). Cloned plasmids were subsequently sequenced to confirm the DNA sequence from promoter regions of WT and mutated TN. 

### 2.5. Luciferase Reporter Assay

The HEK293T cells were seeded on 96-well plates overnight and 200 ng plasmid constructs of WT or mutated TN promoters were individually transfected into HEK293T cells using the PolyJet in vitro DNA Transfection Reagent (SignaGen Laboratories, Rockville, MD, USA) according to the manufacturer’s instructions. The control vector with *Renilla* luciferase (20 ng; Promega) was co-transfected with each constructed firefly luciferase vector containing different promoters of WT or mutated TN to normalize transfection efficiency. The cells were transfected with plasmids for 18 h and then the culture medium was replaced with or without 100 μM DHA conjugated to BSA. Promoter activities were measured using the Dual-Glo Luciferase Assay System (Promega) with a Luminoskan Ascent Microplate Luminometer (Thermo Scientific). The firefly luciferase activities were normalized to *Renilla* luciferase activities within the same samples.

### 2.6. Analysis of Quantitative Real-Time PCR (qPCR)

Total RNA was extracted from tissues or cells using the TRI Reagent (Molecular Research Center Inc., Cincinnati, OH, USA) according to the manufacturer’s instructions. The RNA samples were further digested with a TURBO DNA-free kit (Thermo Scientific) at 37 °C for 30 min to remove residual gDNA contamination followed by reverse transcription with a High-Capacity cDNA Reverse Transcription Kit (Thermo Scientific). qPCR reactions for desired genes were amplified from cDNA using a DyNAmo Flash SYBR Green Kit (Thermo Scientific) and conducted with a CFX96 Real-Time PCR Detection System (Bio-Rad Laboratories, Hercules, CA, USA) with conditions of initial denaturation at 95 °C for 7 min followed by 39 cycles of denaturation at 95 °C for 10 s, annealing/extension at 60 °C for 30 s, with a final extension at 60 °C for 30 s. Primers used for amplification were indicated in Table 3. The threshold cycle (Ct) values were obtained, and relative target gene expression was calculated using a comparative Ct method. The relative values for expression of each target gene were normalized to β-actin expression within the same sample. qPCR amplification efficiency was close to 100%, and all samples were in triplicate. Amplification of specific transcripts was further confirmed by melting curve analysis and agarose gel electrophoresis.

### 2.7. Western Blot Analysis

Total proteins from adipose tissues or PBS-washed cells were extracted using the radioimmunoprecipitation assay buffer (RIPA buffer, Cell Signaling Technology, Danvers, MA, USA). Proteins were quantified using Bradford Reagent (Sigma-Aldrich). Aliquots of proteins (10 μg from tissues extracts or 35 μg from primary-adipocytes extracts) were separated using 12% sodium dodecyl sulfate–polyacrylamide gel electrophoresis and then transferred onto a polyvinylidene fluoride membrane (PerkinElmer, Waltham, MA). TN antibody (Epitomics 3269-1, Abcam, Cambridge, UK), with a 1:10,000 dilution for adipose tissues and a 1:1000 dilution for primary adipocytes, was used as the primary antibody. β-actin antibody (sc-4778, Santa Cruz Biotechnology, Dallas, TX, USA), with a 1:1000 and 1:5000 dilution for adipose tissues and primary adipocytes, respectively, was used as a loading control. Chemiluminescence was detected and the band intensity of target gene TN was quantified and normalized to that of β-actin in the same sample using a UVP BioSpectrum Imaging System (UVP Inc., Upland, CA, USA).

### 2.8. Statistical Analysis

Data were expressed as mean ± standard deviation (SD). Results of two groups were compared by the Student’s *t*-test. Statistical significance among three or more different experimental treatments was determined by one-way analysis of variance (ANOVA). Tukey’s test was used to evaluate differences among means of each group (GraphPad Prism, GraphPad Software, La Jolla, CA, USA). *p* values ≤ 0.05 were considered statistically significant.

## 3. Results

### 3.1. Cloning of Porcine TN

**Figure 1:**
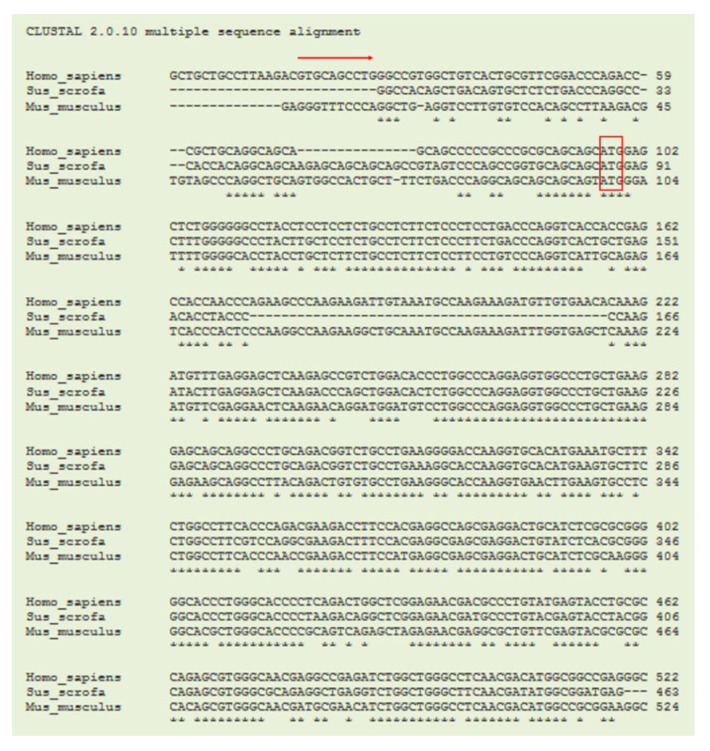
Sequence alignments of TN genes in species. A coding region sequence of the 378-bp porcine (*Sus scrofa*) tetranectin (TN/*CLEC3B*) from cDNA was cloned and aligned to those of humans (*Homo sapiens*) and mice (*Mus musculus*) using the ClustalW program. The arrow indicates the starting exon 1. The box indicates the translation start site. Asterisks indicate bases of homology between all three species.

Currently, the coding sequence (CDS) of porcine gene *CLEC3B* (tetranectin, TN) from the NCBI database is shown as predicted and annotated by automated computational analysis in the porcine genomic database. Therefore, the first step was to obtain the confirmed sequence of porcine TN CDS. Then, the sequence of CDS was used for designing primers for gene expression of porcine TN. The cloned CDS region of porcine TN with 378-bp showed 83% and 90% homology with that of mouse TN/*Clec3b* (NCBI Reference Sequence: NM_011606.2) and that of human TN/*CLEC3B* (NCBI Reference Sequence: NM_003278.2), respectively (Figure 1).

### 3.2. DHA Reduced Expression of TN in Porcine Adipose, Lung and Liver Tissues

**Figure 2:**
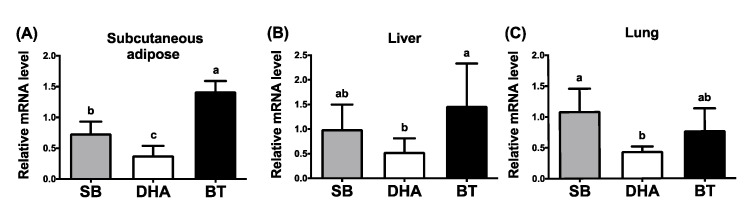
Effects of dietary fats on the expression of TN mRNA in various tissues of weaned piglets. Gene expression of tetranectin (TN/*CLEC3B*) in the (**A**) subcutaneous adipose, (**B**) liver and (**C**) lung tissues of weaned pigs, supplemented with 2% soybean oil (SB), docosahexaenoic acid oil (DHA) or beef tallow (BT) for 30 days, was determined by qPCR and normalized to β-actin in the same sample. Data were expressed as mean ± SD (*n* = 6–8 for each group) and were analyzed by one-way analysis of variance (ANOVA). Different superscript letters (a, b, c) indicate significant difference (*p* ≤ 0.05) determined by Tukey’s multiple comparison test.

**Figure 3:**
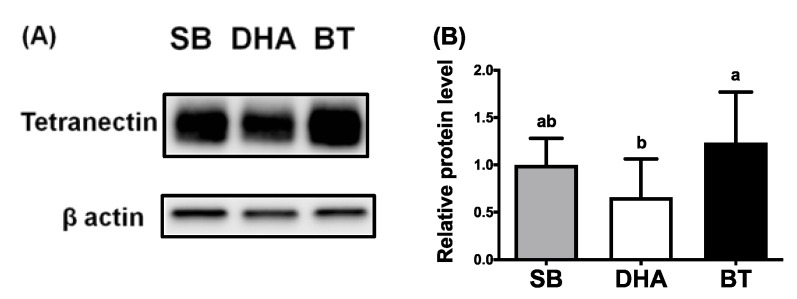
Effects of dietary fats on the protein levels of TN in subcutaneous adipose tissues of weaned piglets. (**A**) Western blot analysis of protein expression for tetranectin (TN) and the loading control of β-actin in adipose tissue of weaned piglets fed with 2% soybean oil (SB), docosahexaenoic acid oil (DHA) or beef tallow (BT) for 30 days. (**B**) Quantification of western blot results for TN protein levels in adipose tissue by dietary supplementation of different fats. Data were expressed as mean ± SD (*n* = 6–8 for each group) and normalized to β-actin expression in the same sample. Data were analyzed by ANOVA. Different superscript letters (a, b) indicate significant difference (*p* ≤ 0.05) determined by Tukey’s multiple comparison test.

Our unpublished data and others [23] indicated that TN expression is positively correlated with adipogenesis, suggesting that TN is an adipogenic adipokine. DHA or fish oil has been shown to reduce adiposity. To examine nutritional modulation by various dietary fats (saturated vs. *n*-6 vs. *n*-3 fatty acids) on TN expression in vivo, the mRNA and protein expression of TN in tissue samples were analyzed from weaned piglets supplemented with 2% BT (enriched in saturated and *n*-9 fatty acids), SB oil (enriched in *n*-6 fatty acids), or DHA oil (an *n*-3 fatty acid). Fatty acid composition in pig feeds, plasma, livers and adipose tissues among treatments of three different diets was analyzed [19,22,24]. Along with reduced plasma levels of triglycerides and cholesterol in pigs [19], gene expression of TN in the porcine subcutaneous adipose tissues was significantly reduced by dietary DHA supplementation compared to adipose from pigs with dietary BT treatments (Figure 2A). A similar diminished trend by DHA supplementation was also observed in the porcine liver and lung tissues (Figure 2B,C). Inhibited protein expression of TN in the porcine subcutaneous adipose tissues by DHA supplementation was revealed (Figure 3A,B). These results indicate that both mRNA and protein expression of TN were attenuated by dietary DHA oils in pigs, suggesting that adipogenic TN was a potential target mediating the anti-obesity effect of DHA in pigs.

### 3.3. DHA Reduced Expression of TN in Porcine Adipocytes

**Figure 4:**
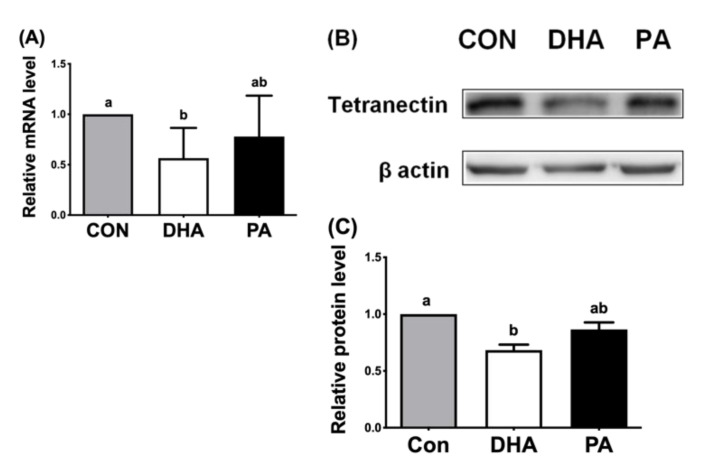
Effects of fatty acids on TN expression in porcine mature adipocytes in vitro. (**A**) Gene expression of tetranectin (TN/*CLEC3B*), normalized to β-actin expression, in the mature adipocytes, differentiated from adipose stem cells/pre-adipocytes. Treatments were BSA solutions with the vehicle control (ethanol; CON), 100 μM of docosahexaenoic acids (DHA) or 100 μM of saturated palmitic acids (PA) for 24 h and then extracted and determined by qPCR. (**B**) Western blot of TN and β-actin protein expression. (**C**) Quantification of western blots for TN protein expression among fatty acid treatments in mature adipocytes. Data were expressed as mean ± SD (*n* = 6–8 for each group) and normalized to β-actin expression in the same sample. Data were analyzed by ANOVA. Different superscript letters (a, b) indicate significant difference (*p* ≤ 0.05) determined by Tukey’s multiple comparison test.

To confirm the in vivo inhibitory effect of DHA on TN gene expression, mature adipocytes differentiated from porcine adipose stem cells within the stromal/vascular fraction were examined for DHA effects attenuating TN. Mature adipocytes treated with DHA for 24 h had decreased mRNA (Figure 4A) and protein expression (Figure 4B,C) of TN compared to saturated PA-supplemented adipocytes or controls. These results showed that mRNA and protein expression of porcine TN were significantly reduced in the DHA-treated adipocytes in an autologous fashion to fatty acid effects in vitro and in vivo.

### 3.4. Cloning of Porcine TN Promoter and Identification of Putative Binding Sites for Transcription Factors on Porcine TN Promoter

**Figure 5:**
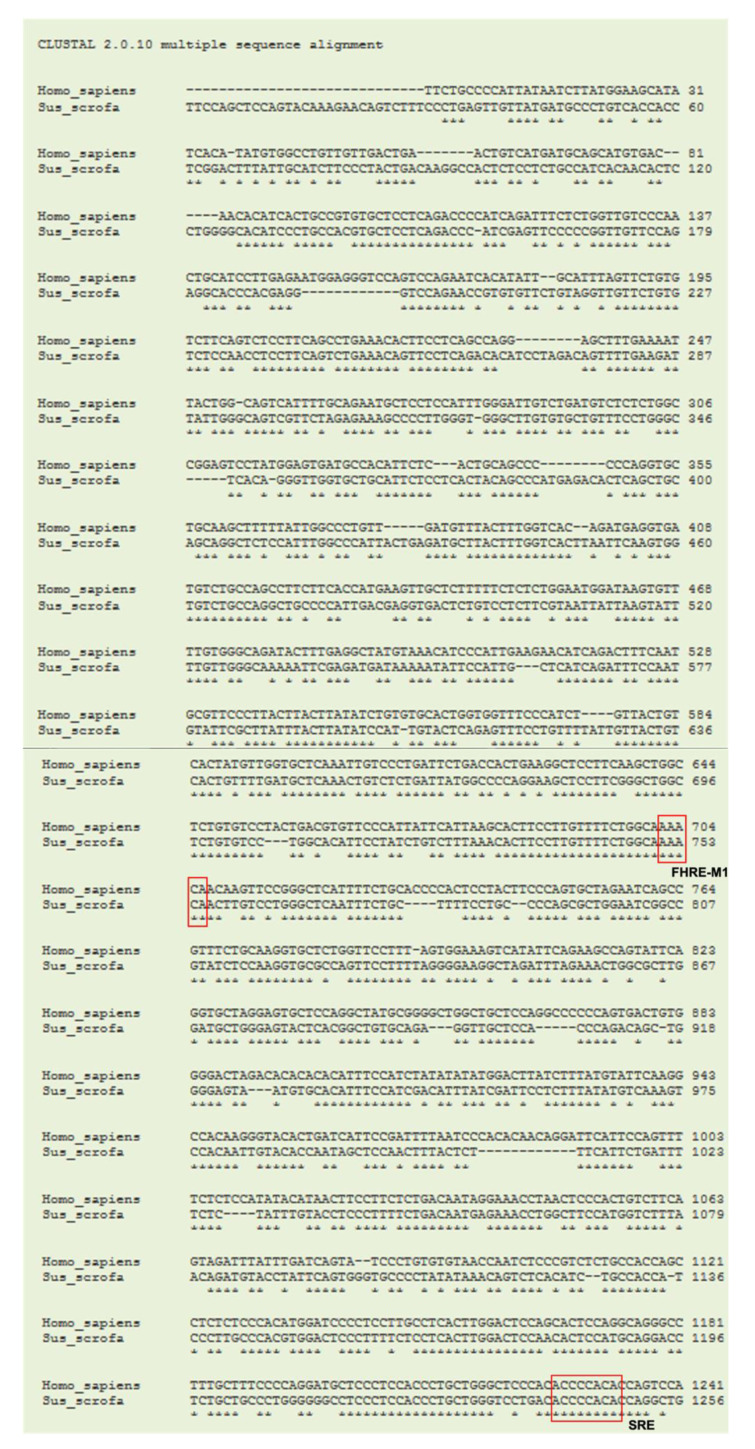

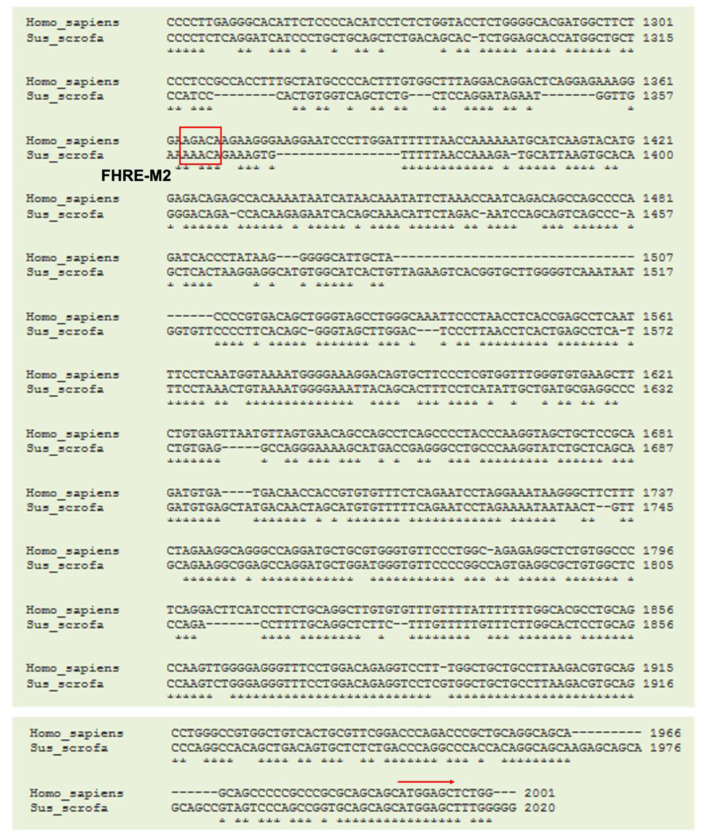
Sequence alignments of the putative porcine TN promoter by the ClustalW program. A 1956-bp sequence of putative porcine tetranectin (TN/*CLEC3B*) promoter was cloned and aligned to that of the human sequence. The porcine sequence had 65% homology to the human TN promoter. Red boxes indicate consensus elements of binding sites, sterol regulatory elements (SRE) for SREBP-1c and forkhead response elements (FHRE) for FoxO in the pig and human sequences. The red arrow indicates the translation initiation site.

To uncover the underlying mechanism of transcriptional regulation on inhibited TN expression by DHA supplementation, a putative porcine TN promoter (1956-bp) was cloned and compared with the human TN promoter (NC_000003.11). A 65% homology was revealed between porcine and human TN promoters (Figure 5). Moreover, putative binding sites for transcription factors, one SRE site for SREBP-1c and two FHRE sites for FoxO, were predicted on the porcine TN promoter, and these binding sites were conserved in both human and porcine TN promoters (Figure 5).

### 3.5. DHA Repressed Expression of FOXO and SREBP-1c in Porcine Adipose Tissues and Adipocytes

**Figure 6:**
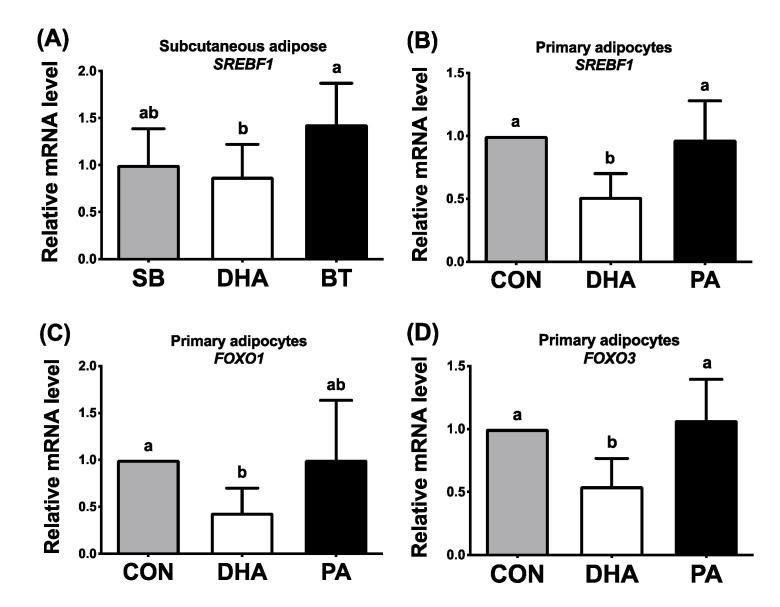
Expression of porcine SREBP-1c, FoxO1 and FoxO3 in DHA-supplemented adipose tissues or primary adipocytes. (**A**) Expression of sterol regulatory element-binding protein 1c (SREBP-1c/*SREBF1*) in subcutaneous adipose tissues of weaned pigs, supplemented with 2% soybean oil (SB), docosahexaenoic acid oil (DHA) or beef tallow (BT) for 30 days. The mRNA was determined by qPCR (*n* = 6–8 for each group). Expression of SREBP-1c/*SREBF1* (**B**), forkhead box O1 (FoxO1) (**C**), and FoxO3 (**D**) in mature adipocytes, differentiated from adipose stem cells/pre-adipocytes. Cells were treated with the vehicle control (ethanol; CON), 100 μM of docosahexaenoic acids (DHA) or 100 μM of saturated palmitic acids (PA) for 24 h and then the mRNA sequence was determined by qPCR (*n* = 7 for each group). Data were expressed as mean ± SD and normalized to β-actin expression in the same sample. Data were analyzed by ANOVA. Different superscript letters (a, b) indicate significant difference (*p* ≤ 0.05) determined by Tukey’s multiple comparison test.

Binding sites for SREBP-1c and FoxO on the TN promoter suggest that SREBP-1c and/or FoxO could transcriptionally regulate TN expression. To elucidate whether DHA inhibits TN expression through SREBP-1c and FoxO, expression of the major related transcription factors SREBP-1c, FoxO1 and FoxO3 was determined under DHA supplementation. Compared with the BT group, dietary DHA supplementation in pigs inhibited expression of adipose *SREBF1* (Figure 6A) and adipose *FOXO1* and *FOXO3* in our previous studies [19]. Supplementation of porcine primary adipocytes (differentiated from adipose stem cells) with DHA for 24 h also inhibited expression of *SREBF1* (Figure 6B), *FOXO1* (Figure 6C), and *FOXO3* (Figure 6D) compared to PA-treated primary adipocytes. These results indicate that DHA administration hampers expression of the transcription factors SREBP-1c, FoxO1, and FoxO3, which could result in inhibited transcription of TN expression. 

### 3.6. Transcriptional Suppression of TN Expression by DHA Through SREBP-1c and FoxO in HEK293T Cells

**Figure 7:**
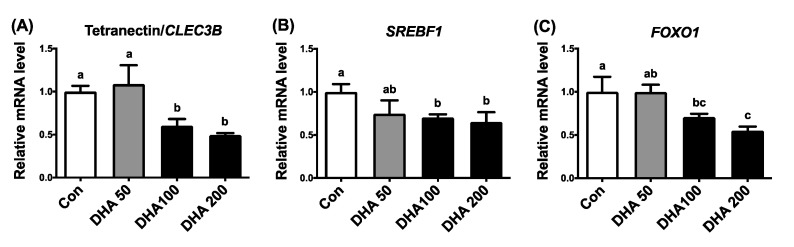
Effects of DHA on expression of TN, SREBP-1c and FoxO1 in human HEK293T cells. Expression of (**A**) tetranectin (TN/*CLEC3B*), (**B**) sterol regulatory element-binding protein 1c (SREBP-1c/*SREBF1*) and (**C**) forkhead box O1 (FoxO1) was determined by qPCR in human HEK293T cells. HEK293T cells were cultured and supplemented with various concentrations (0 = Con, 50, 100, 200 μM) of docosahexaenoic acids (DHA) for 24 h (*n* = 3). Data were expressed as mean ± SD and normalized to β-actin expression in the same sample. Data were analyzed by ANOVA. Different superscript letters (a, b, c) indicate significant difference (*p* ≤ 0.05) determined by Tukey’s multiple comparison test.

**Figure 8:**
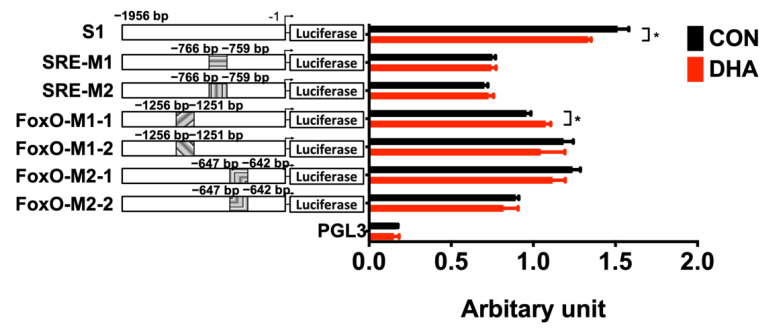
Identification of DHA-modulated response elements on TN promoter in HEK293T cells. Wild type (S1) or mutated promoter activities for gene tetranectin (TN/*CLEC3B*) were determined in HEK293T cells treated with 100 μM of DHA for 24 h by co-transfecting firefly and *Renilla* luciferase vectors. The TN promoter had one predicted mutation site for sterol regulatory element (SRE) and two mutation sites for forkhead response elements (FHRE). Seven clones were generated and cloned into each firefly luciferase vector including one 1956-bp of wild-type TN promoter (S1), two different clones (M1 and M2) within the same mutated location of SRE, and two clones of mutated FHRE at two dispersed locations (M1-1, M1-2, M2-1, and M2-2). The values of relative light units (RLU) were normalized to correct for transfection efficiency using the *Renilla* luciferase activity. Data were expressed as means ± SD (*n* = 3) with arbitrary units and analyzed by Student’s *t*-test. Asterisks indicate statistical significances, *p* ≤ 0.05. SRE-M1: clone 1 of TN promoter with mutated SRE (−759 to −766); SRE-M2: clone 2 of TN promoter with mutated SRE (−759 to −766); FoxO-M1-1: clone 1 of TN promoter with mutated FHRE at first mutation site (−1251 bp to −1256); FoxO-M1-2: clone 2 of TN promoter with mutated FHRE at first mutation site (−1251 bp to −1256); FoxO-M2-1: clone 1 of TN promoter with mutated FHRE at second mutation site (−642 bp to −647); FoxO-M2-2: clone 2 of TN promoter with mutated FHRE at second mutation site (−642 bp to −647).

To elucidate the transcriptional regulation of DHA on TN expression, we turned to human HEK293T cells due to their higher transfection efficiency than other cell types. Moreover, binding elements for the transcription factors SREBP-1c and FoxO, on TN promoter, are also conserved in humans and pigs (Figure 5). First, phenocopying effects of attenuated TN expression in porcine adipose tissues and primary adipocytes by supplementation with DHA needed to be evaluated in human HEK293T cells. Our previous study demonstrated, by lactate dehydrogenase (LDH) assay, that HEK293T cells can stand supplementation of DHA concentrations up to 200 μM without cytotoxicity [25]. Supplementing HEK293T cells with 100 or 200 μM of DHA reduced not only TN/*CLEC3B* expression (Figure 7A), but also *SREBF1* (Figure 7B) and *FOXO1* (Figure 7C) expression compared to the control group. To evaluate the transcription activation in the porcine TN promoter, a 1956-bp wild-type porcine TN promoter (S1) was cloned. Putative binding sites for transcription factors on the porcine TN promoter were predicted by TFsitescan and revealed one SRE for SREBP-1c, at −766 to −759, and two FHRE for FoxO, at −1256 to −1251 and at −647 to −642 (Figure 5). The 100 μM DHA treatment reduced the promoter activities of TN in HEK293T cells transiently transfected with the promoter constructs and measured by luciferase activity (Figure 8). The inhibition of wild-type TN promoter (S1) activity by DHA supplementation was abolished in HEK293T cells with all mutated constructs of TN promoters containing either mutated binding sites, SRE (SRE-M1, SRE-M2) for SREBP-1c, or FHRE (FoxO-M1-1, FoxO-M1-2, FoxO-M2-1, FoxO-M2-2) for FoxO. In contrast, the TN promoter activity was even activated by DHA treatments in HEK293T cells with mutated TN promoter containing one mutated FHRE (FoxO-M1-1) (Figure 8). These results suggest that transcriptional attenuation of TN by DHA could occur partly through inhibition of the binding of SREBP-1c and FoxO to the TN promoter. 

## 4. Discussion

The current study, to our knowledge, is the first to (1) confirm the coding sequence and the promoter region for porcine TN in pig lung and adipose tissues, (2) unveil nutritional modulation of TN expression inhibited by an *n*-3 PUFA, DHA in human cells and pig tissues and (3) identify putative binding sites for transcription factors on the porcine TN promoter that is regulated by DHA supplementation.

### 4.1. Negative Regulation of TN by DHA

Dietary fatty acid is an important factor associated with burgeoning global obesity, especially the imbalanced ratio of *n*-6 to *n*-3 PUFA [26]. With respect to fatty acid regulation on TN expression, this study is the first to show that, compared to BT diets enriched in saturated fatty acids and oleic acids, TN expression is inhibited by diets enriched in an *n*-3 PUFA, DHA in porcine adipose, lung, and liver tissues. This inhibition of DHA by an *n*-3 PUFA diet was not evident in the case of an *n*-6 PUFA diet derived from soybean oil in vivo. Therefore, it seems that repression of TN expression is only specifically regulated by the *n*-3 fatty acid, DHA, but not by *n*-6, *n*-9, or saturated fatty acids.

Our previous studies demonstrated that, compared with beef tallow supplementation, dietary supplementation with 2% algal DHA oil for 30 days [19] or for 18 days [24] altered fatty acid composition and metabolism genes in livers and adipose tissues of weaned pigs. Plasma triglycerides and adipose tissue mass also may be reduced by dietary DHA. This reduction may occur through the promotion of lipolysis [17,25], or through an interaction with several transcription factors and nuclear receptors that take part in adipocyte differentiation, including C/EBP, SREBP, PPAR, and LXR. 

In addition to these well-known transcription factors and nuclear receptors, the current study showed inhibited expression of the transcription factors SREBP-1c, FoxO1, and FoxO3 in pig primary adipocytes and human HEK293T cells, which is consistent with our previous results observed in vivo [19] and in vitro [27], suggesting conserved regulation of TN by DHA in humans and pigs.

The SREBP family is composed of three proteins: SREBP-1a, SREBP-1c and SREBP-2. SREBP belong to the basic helix-loop-helix transcription factor family and are necessary for the insulin actions on lipid and glucose metabolism. SREBP-1a and SREBP-1c are important players in upregulating numerous genes related to fatty acid biogenesis, whereas SREBP-2 mainly participates in cholesterol metabolism [28]. SREBP-1c gene expression is upregulated in early adipocyte differentiation and can regulate PPARγ gene expression through the production of endogenous PPARγ ligands [18]. In addition, SREBP-1c is able to directly transactivate the C/EBPβ promoter [29]. 

Repression of SREBP-1c by DHA accelerates the degradation of SREBP-1c mRNA, leading to downregulation of the expression of fatty acid synthetase (FAS). Therefore, the DHA inhibitory effect on lipogenesis may be mainly mediated by SREBP-1c mRNA decay [30]. Moreover, *n*-3 PUFA can not only promote lipolysis but also downregulate several lipogenic genes such as FAS, acetyl-CoA carboxylase, and stearoyl-CoA desaturase 1 through a SRE binding site for SREBP-1c [31,32]. The current study showed that transcriptional activation of TN could act through the binding site of SRE for SREBP-1c on the porcine TN promoter. However, the precise mechanism of SREBP-1c in the regulation of tetranectin requires further investigation.

Forkhead box O proteins are also essential players for lipid metabolism. It has been reported that FoxO1 is the dominant FoxO isoform in murine liver and white adipose tissues [33]. Moreover, FoxO are negatively regulated by insulin, and increase during adipogenesis. This regulation acts through dephosphorylation and translocation from cytoplasm to the nucleus of FoxO1 in the adipogenic 3T3-F442A cell line [34] 

FoxO are also involved in obesity-related diseases. Overexpression of FoxO1 in the hypothalamus and pancreas accounts for obesity, insulin resistance, glucose intolerance and hyper-triglyceridemia [35]. Treatment with the PPARγ agonist, rosiglitazone, results in an improvement in insulin sensitivity in FoxO1 haplo-insufficient mice [36]. Transgenic mice with an adipose tissue-specific mutation of FoxO1 showed improved glucose tolerance and insulin sensitivity with smaller-sized adipocytes and increased adiponectin when fed with a high-fat diet [37]. These results indicate that the reduction in FoxO ameliorates the state of obesity-related diseases [38]. Our previous study showed that dietary DHA reduces not only FoxO1 and FoxO3 expression in porcine adipose and liver tissues and adipocytes, but also FoxO target genes pertinent to gluconeogenesis and triglyceride synthesis [19]. This study also showed that DHA supplementation restrained expression of FoxO1 and FoxO3 in pig adipocytes and human kidney HEK293T cells, suggesting that FoxO are potential therapeutic targets for DHA in ameliorating metabolic syndromes.

### 4.2. TN Promoter Regulation by DHA

The luciferase reporter assay for the porcine TN promoter with wild-type binding sites, mutated binding sites of SRE for SREBP-1c, or mutated binding sites of FHRE for FoxO, revealed that attenuated transactivation of porcine TN by DHA supplementation was abolished in the SRE- or FHRE-mutated TN promoter, implying that the inhibitory effect of DHA on TN may be partly due to transcription factors, specifically SREBP-1 and/or FoxO. In addition, a previous study showed that overexpressing a constitutive form of nuclear FoxO1 increases gene expression of SREBP-1c in mouse livers [39]. Our previous studies showed that DHA supplementation inhibits expression of FoxO1/FoxO3 [19] and SREBP-1c [24] in porcine liver and adipose tissues both in vivo and in vitro. Therefore, FoxO1 and SERBP-1c appear to act synergistically on TN promoter to transactivate TN expression, and DHA supplementation disrupts this activation. Moreover, the current study revealed that putative binding sites of one SRE for SREBP-1c and two FHRE for FoxO were conserved in both human and porcine TN promoters, suggesting that transcription activation on porcine and human TN promoter could be similar (Figure 5). However, comparison of parallel phenocopies in the TN promoter regulation of humans and pigs awaits further investigation.

### 4.3. Implication of TN in Adipogenesis

This study revealed that TN expression in porcine adipose tissues and primary adipocytes is congruent with our previous preliminary results, indicating that TN is an adipokine that is secreted by human differentiated mature adipocytes, increased during adipogenesis of human adipocytes and positively correlated with body mass index of human subjects. TN is also increased in obese mice when fed a high-fat diet for 16 weeks, and overexpression of TN in human adipocytes results in lipid accumulation and elevated lipogenic gene expression [20]. Therefore, TN may play an important role in adipogenesis in humans, mice and pigs.

Our preliminary results [20] and other studies showed that, by 2-D LC-MS/MS or quantitative proteomics, conditioned media from adipocyte differentiation processes or mature adipocytes contain secreted TN in humans [40], mice [41,42,43], and rats [44]. This suggests that TN is an adipokine related to adipocyte adipogenesis, although one study showed that TN expression is downregulated after adipocyte differentiation [23]. Another fat-tissue distribution analysis revealed that TN is highly expressed in human subcutaneous [45] and mouse visceral [46] adipose tissues.

Further functional analysis with the addition of an anti-TN antibody for TN blockage during adipocyte differentiation showed strong abolishment of adipocyte differentiation [42]. In contrast, exogenous tetranectin supplementation enhances adipocyte differentiation, which requires exon 3 of the plasminogen binding domain of TN [43]. Therefore, TN appears to be an adipogenic adipokine that is related to the augmentation of adipocyte differentiation.

Moreover, TN expression is increased in mouse adipocytes with the insulin-resistance state induced by a high-glucose plus insulin medium [41], and in insulin-resistant Swedish male individuals, compared to the control group [47]. Therefore, reducing expression of TN could be a beneficial approach to improve obesity-induced insulin resistance or related metabolic syndromes.

Although we demonstrated in the study that the adipogenic TN was impeded by an *n*-3 fatty acid diet of DHA supplementation, both in pigs in vivo and in humans/pigs in vitro, there are still some limitations. We showed that DHA inhibited tetranectin expression in human kidney HEK293T cells under in vitro condition, but data are not available on whether DHA or fish oil supplementation also inhibits tetranectin expression in metabolic tissues of human clinical subjects. Furthermore, we pinpointed certain transcription factors (SREBP-1c and FoxO) involved in the transcription of tetranectin expression. Nonetheless, related hormonal regulation modulating SREBP-1c or FoxO signaling on tetranectin expression needs to be uncovered. Finally, does the adipokine TN synergistically augment or mitigate functions of other adipokines, such as adiponectin, resistin, or leptin? These interesting but unexplored questions remain to be revealed.

## 5. Conclusions

Taken together, the newly identified adipokine, adipogenic tetranectin, was inhibited by DHA supplementation in adipose tissues in vivo, and in primary adipocytes and human HEK293T cells in vitro. This inhibition was accompanied by reduced expression of SREBP-1c, FoxO1 and FoxO3. Promoter analysis showed that this DHA-attenuated transactivation of the TN promoter was dependent on abrogating transcription binding sites for SERBP-1c and FoxO. Therefore, the beneficial effect of DHA to reduce TN expression may partly result from down-regulating SREBP-1c and FoxO, which could shed new light on preventive and therapeutic strategies for obesity-associated diseases.

## Figures and Tables

**Table 1:** List of primers for cloning coding sequence (CDS) of porcine gene *CLEC3B* (tetranectin, TN) from porcine cDNA library.

Gene	Sense (5′ → 3′)	Antisense (5′ → 3′)	Temp	Accession Number
*CLEC3B*, clone 1 (536–737)	CACCAAGGTGCACATGAAGT	CTCATCCGCCATATCGTTG	60 ℃	XM_005669451.3
*CLEC3B*, clone 2 (377–602)	TGAGACACCTACCCCCAAGA	CTCGCTCGCCTCGTGGAAAGTC	58 ℃	XM_005669451.3
*CLEC3B*, clone 3 (166–408)	GTGCTCTCTGACCCAGGCCCA	CTCGCTCGCCTCGTGGAAAGTC	58 ℃	XM_005669451.3

**Table 2:** List of primers for the putative promoter region of porcine tetranectin (TN/*CLEC3B*).

Promoter	Sense (5′ → 3′)	Antisense (5′ → 3′)
TN-S1 (wild-type)	GCGCGGTACCCTGTCACCACCTCGGACTTT	GCGCAAGCTTGCTGCTGCACCGGCTGGGA
TN-SRE site 1-mutant 1 (SRE-M1), first half part	GCGCGGTACCCTGTCACCACCTCGGACTTT	CAGCCTGGTTTTGGGTGTCAGGA
TN-SRE site 1-mutant 1 (SRE-M1), second half part	TCCTGACACCCAAAACCAGGCTG	GCGCAAGCTTGCTGCTGCACCGGCTGGGA
TN-SRE site 1-mutant 2 (SRE-M2), first half part	GCGCGGTACCCTGTCACCACCTCGGACTTT	CAGCCTGGTGTGGTTTGTCAGGA
TN-SRE site 1-mutant 2 (SRE-M2), second half part	TCCTGACAAACCACACCAGGCTG	GCGCAAGCTTGCTGCTGCACCGGCTGGGA
TN-FHRE/FoxO site 1-mutant 1 (FoxO-M1-1), first half part	GCGCGGTACCCTGTCACCACCTCGGACTTT	GCAGAAATTGAGCCCAGGACAAGTTGCCTTGCCAG
TN-FHRE/FoxO site 1-mutant 1 (FoxO-M1-1), second half part	CTGGCAAGGCAACTTGTCCTGGGCTCAATTTCTGC	GCGCAAGCTTGCTGCTGCACCGGCTGGGA
TN-FHRE/FoxO site 1-mutant 2 (FoxO-M1-2), first half part	GCGCGGTACCCTGTCACCACCTCGGACTTT	GCAGAAATTGAGCCCAGGACAAGTCGTCTTGCCAG
TN-FHRE/FoxO site 1-mutant 2 (FoxO-M1-2), second half part	CTGGCAAGACGACTTGTCCTGGGCTCAATTTCTGC	GCGCAAGCTTGCTGCTGCACCGGCTGGGA
TN-FHRE/FoxO site 2-mutant 1 (FoxO-M2-1), first half part	GCGCGGTACCCTGTCACCACCTCGGACTTT	CACTTTCTGCCTTTCAACCATTCTATCCTGGAGC
TN-FHRE/FoxO site 2-mutant 1 (FoxO-M2-1), second half part	GCTCCAGGATAGAATGGTTGAAAGGCAGAAAGTG	GCGCAAGCTTGCTGCTGCACCGGCTGGGA
TN-FHRE/FoxO site 2-clone 2 (FoxO-M2-2), first half part	GCGCGGTACCCTGTCACCACCTCGGACTTT	CACTTTCTATCTTTCAACCATTCTATCCTGGAGC
TN-FHRE/FoxO site 2-clone 2 (FoxO-M2-2), second half part	GCTCCAGGATAGAATGGTTGAAAGATAGAAAGTG	GCGCAAGCTTGCTGCTGCACCGGCTGGGA

SRE- or FHRE-mutated constructs are derived from wild-type TN (TN-S1) promoter.

**Table 3:** List of primers for gene expression by quantitative real-time PCR analysis.

Gene	Sense (5′ → 3′)	Antisense (5′ → 3′)	Accession Number
porcine *CLEC3B* (tetranectin, TN)	GAGGTGGCCCTGCTGAAGGA	CTCGCTCGCCTCGTGGAAAGTC	XM_005669451.2
porcine *ACTB* (β-actin)	ATCGTGCGGGACATCAAG	AGGAAGGAGGGCTGGAAG	AY550069.1
porcine *SREBF1* (SREBP-1c)	GCGACGGTGCCTCTGGTAGT	CGCAAGACGGCGGATTTA	AF102873.1
porcine *FOXO1*	AAGAGCGTGCCCTACTTCAA	TTCCTTCATTCTGCACACGA	NM_214014.2
porcine *FOXO3*	GGCTGGAAGAACTCTATC	GTACTTGTTGCTGTTGTC	NM_001135959.1
human *CLEC3B* (tetranectin, TN)	GAGCTCTGGGGGGCCTAC	CTTGGCATTTACAATCTTCTTGG	NM_003278.2
human *ACTB* (β-actin)	AAGATCAAGATCATTGCTCCTCC	CGGACTCGTCATACTCCTG	NM_001101.3
human *SREBF1* (SREBP-1c)	CTGTTGGTGCTCGTCTCCTTGG	CAGCAGGTGACGGATGAGGTTC	NM_001005291.2
human *FOXO1*	CGGAATGACCTCATGGATGGA	TAAGTGTAACCTGCTCACTAACCC	NM_002015.3

## Data Availability

Vectors are supplied as requested.
